# Alantolactone attenuates high-fat diet-induced inflammation and oxidative stress in non-alcoholic fatty liver disease

**DOI:** 10.1038/s41387-024-00300-7

**Published:** 2024-06-10

**Authors:** Jiong Wang, Yucheng Jiang, Leiming Jin, Chenchen Qian, Wei Zuo, Jianjun Lin, Longteng Xie, Bo Jin, Yanni Zhao, Lijiang Huang, Yi Wang

**Affiliations:** 1 Joint Research Center on Medicine, the Affiliated Xiangshan Hospital of Wenzhou Medical University, Ningbo, 315700 Zhejiang China; 2 Chemical Biology Research Center, School of Pharmaceutical Sciences, Wenzhou Medical University, Wenzhou, Zhejiang China; 3 School of Pharmacy, Hangzhou Normal University, Hangzhou, Zhejiang China

**Keywords:** Fats, Obesity

## Abstract

**Background:**

Nonalcoholic fatty liver disease (NAFLD) is a chronic disease with an increasing incidence, which can further develop into liver fibrosis and hepatocellular carcinoma at the end stage. Alantolactone (Ala), a sesquiterpene lactone isolated from Asteraceae, has shown anti-inflammatory effects in different models. However, the therapeutic effect of Ala on NAFLD is not clear.

**Methods:**

C57BL/6 mice were fed a high-fat diet (HFD) to induce NAFLD. After 16 weeks, Ala was administered by gavage to observe its effect on NAFLD. RNA sequencing of liver tissues was performed to investigate the mechanism. In vitro, mouse cell line AML-12 was pretreated with Ala to resist palmitic acid (PA)-induced inflammation, oxidative stress and fibrosis.

**Results:**

Ala significantly inhibited inflammation, fibrosis and oxidative stress in HFD-induced mice, as well as PA-induced AML-12 cells. Mechanistic studies showed that the effect of Ala was related to the induction of Nrf2 and the inhibition of NF-κB. Taken together, these findings suggested that Ala exerted a liver protective effect on NAFLD by blocking inflammation and oxidative stress.

**Conclusions:**

The study found that Ala exerted a liver protective effect on NAFLD by blocking inflammation and oxidative stress, suggesting that Ala is an effective therapy for NAFLD.

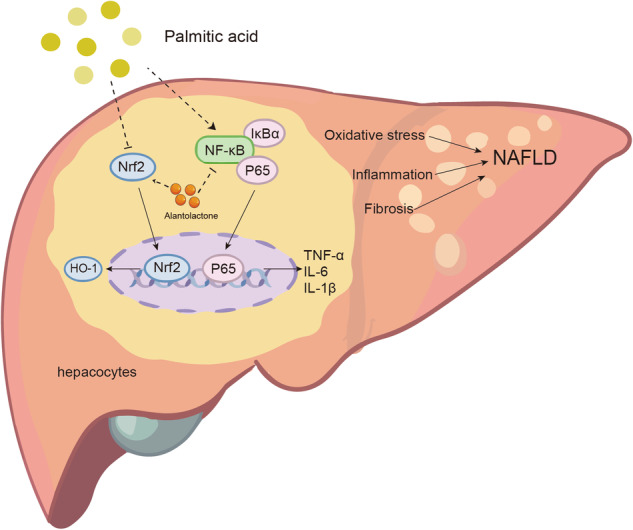

## Introduction

In recent years, obesity and its related diseases have become a global issue [1]. Among them, non-alcoholic fatty liver disease (NAFLD) is closely related to obesity, and its prevalence is continuously increasing worldwide [2]. NAFLD is characterized by hepatic steatosis, but it also includes non-alcoholic steatohepatitis (NASH), which is characterized by liver inflammation, hepatocellular injury and fibrosis. This highlights the potential progression of the disease [3]. The pharmacological treatment of NAFLD or NASH remains an unmet clinical need. To date, no drugs have been approved by the FDA for the treatment of NASH [4].

So far, the mechanisms underlying NAFLD remain unclear, but chronic inflammation and oxidative stress play crucial roles in the development and progression of NAFLD [5]. The pathological progression of NAFLD initially follows a “three-hit” process, which includes steatosis, lipotoxicity, and inflammation [6]. On the one hand, steatosis is characterized by increased activation of the transcription factor NF-κB (nuclear factor kappa B) through the upstream activation of IKKβ (inhibitor of kappa B kinase β). Activation of NF-κB induces the production of pro-inflammatory mediators such as TNF-α (tumor necrosis factor-alpha), IL-6 (interleukin-6), and IL-1β (interleukin-1 beta) [4]. On the other hand, excessive fat accumulation in the liver leads to lipotoxicity, resulting in organelle dysfunction, primarily involving mitochondrial impairment and endoplasmic reticulum stress, leading to the generation of reactive oxygen species (ROS) [7, 8]. Excessive ROS leads to mitochondrial damage, hepatocyte apoptosis, and induces lipid peroxidation, resulting in inflammation and fibrogenesis [9, 10]. Under the drive of inflammation and oxidative stress, the liver suffers more severe damage, leading to the development of end-stage liver fibrosis and cirrhosis.

Alantolactone (Ala), a natural compound extracted from a traditional Chinese medicine *Inula helenium L*., has the potential to treat a variety of diseases [11]. Ala has anti-tumor, antibacterial, neuroprotective and other pharmacological effects, the most important of which is anti-inflammatory [12]. For example, in streptozotocin-induced diabetic mice, Ala inhibited high glucose-induced proinflammatory cytokine production by inhibiting the NF-κB pathway [13]. In addition, Ala inhibited CSE-induced inflammation, apoptosis, and oxidative stress by regulating the NF-κB and Nrf2/HO-1 axis [14]. However, it remains unknown whether Ala exhibits therapeutic affects in the treatment of NAFLD.

Our research aimed to investigate the hepatoprotective effects of Ala on NAFLD. Our study indicated that Ala significantly alleviated hepatic pathological changes induced by a HFD through the inhibition of inflammatory responses and oxidative stress. These findings suggested that Ala might be a potential candidate drug for the treatment of NAFLD.

## Materials and methods

### Reagents

**Fig. 1:**
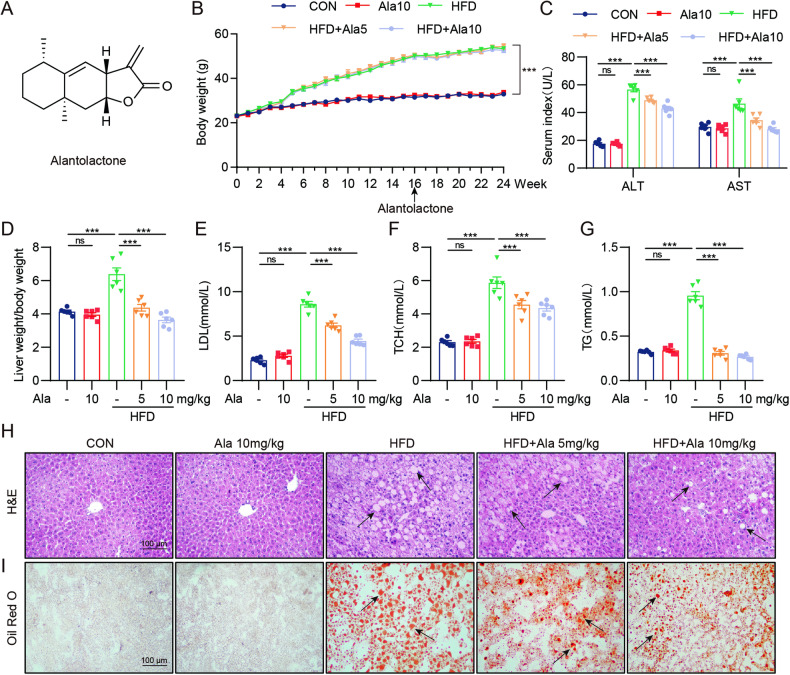
Ala attenuates high-fat diet (HFD)-induced liver dysfunction. **A** Chemical structure of Ala. **B** Body weight levels in mice. Serum levels of liver function markers AST and ALT (**C**), and lipids (**D**–**G**). **H** Representative H&E-stained images of liver tissues [scale bar = 100 μm]. **I** Representative images showing Oil red O staining of liver tissues [scale bar = 100 μm]. Data are expressed as Mean ± SEM, *n* = 6 per group. ***p < 0.001, ns not significant.

Alantolactone was purchased from Macklin (Shanghai, China) and dissolved in DMSO for experiments in vitro and in 0.5% CMC-Na for studies in vivo. The chemical structure of Alantolactone is shown in Fig. 1A. Antibodies against inhibitor of IκB-α, NF-κB P65 subunit, p-P65 and GAPDH were purchased from Cell Signaling Technology (Danvers, MA, USA). Anti-TGF-β1 antibody and anti-Lamin B were acquired from Abcam (Cambridge, MA, USA). Antibodies against COL1, α-SMA, Nrf-2, HO-1 and Keap1 were purchased from Proteintech (Wuhan, China). Total cholesterol (TCH), triglyceride (TG), low-density lipoprotein-cholesterol (LDL-C), alanine aminotransferase (ALT), and aspartate aminotransferase (AST) were purchased from Nanjing Jiancheng Bioengineering Institute (Nanjing, China). Total superoxide dismutase assay kit (SOD (superoxide dismutase)) and lipid Peroxidation MDA assay kit (MDA (Malondialdehyde)) were purchased from Shanghai Beyotime Biotechnology (Shanghai, China). Masson’s trichrome staining kit, hematoxylin and eosin (H&E) staining kit, Sirius Red staining kit and Oil red O staining kit were purchased from Solarbio Life Sciences (Beijing, China). Sodium palmitate (PA) and bovine serum albumin (BSA) were purchased from Sigma-Aldrich (St. Louis, MO, USA).

### Cell culture

The AML mouse-derived liver cell line was obtained from the Shanghai Institute of Biochemistry and Cell Biology (cat# SCSP-550, Shanghai, China). The cells were cultured in DMEM-F12 medium (Gibco, Eggenstein, Germany) supplemented with 10% FBS (Gibco, Eggenstein, Germany), 100 U/mL penicillin/streptomycin (Gibco, Eggenstein, Germany), 0.1% insulin-transferrin-selenium (ITS, Sigma, Burligton USA), and 40 ng/mL dexamethasone (Sigma, Burligton USA). The culture was maintained in a humidified incubator at 37 °C with 5% CO2.

### Animals

Male C57BL/6 mice weighing 18–20 g were sourced from the Animal Center of Wenzhou Medical University. All animal care and experimental procedures were conducted in accordance with the guidelines set forth by the Wenzhou Medical University Animal Policy and Welfare Committee (Approval Document No. wydw2019-0145). The mice were housed at a temperature of 22 °C with a relative humidity of 60% and provided with a standard rodent diet and purified water.

Mice were randomly divided into 5 groups: (i) untreated mice receiving 0.5% CMC-Na solution (CON, *n* = 6) ; (ii) untreated mice receiving 10 mg/kg/2d Ala (Ala 10 mg/kg; *n* = 6) ; (iii) high-fat diet mice receiving 0.5% CMC-Na solution (HFD; *n* = 6) ; (iv) high-fat diet mice treated of 5 mg/kg/2d Ala (HFD + Ala 5 mg/kg; *n* = 6) ; and (v) high-fat diet mice treated of 10 mg/kg/2d Ala (HFD + Ala 10 mg/kg; *n* = 6).

CON group mice and Ala 10 mg/kg group mice were fed with the low-fat diet. The LFD comprised 10 kcal% fat, 20 kcal% protein, and 70 kcal% carbohydrates (MediScience Yangzhou, China; cat. #MD12031). The HFD consisted of 60 kcal% fat, 20 kcal% protein, and 20 kcal% carbohydrates (cat. #MD12033). The mice were weighed weekly. After 16 weeks of continuous feeding, Ala and 0.5% CMC-Na were administered as oral gavage once every 2 days for another 8 weeks. At the end of treatment, mice were sacrificed under sodium pentobarbital (intraperitoneal [i.p.] injection of 0.2 mL sodium pentobarbital at 100 mg/mL) for anesthesia and pain mitigation. Liver tissues and serum were harvested.

### MTT assay

MTT powder (cat. no. M8180, Solarbio; Beijing, China) was dissolved in phosphate-buffered saline. AML-12 cells (4000 per well) were seeded in 96-well plates. After cell adherence, Ala was added to the wells at various doses (0.2–50 μM), followed by incubation for 48 h. Next, MTT was added to each well (1 mg/mL) and incubated for 4 h. The formazan crystals were then dissolved in dimethyl sulfoxide (DMSO; 150 μL/well), and absorbance was examined at 490 nm using SpectraMax M5 microplate reader (Molecular Devices, CA, USA).

### Histological analyses

Liver tissues of each group were fixed with 4% paraformaldehyde, embedded in paraffin wax and sliced into 5 μm sections. H&E, Sirius red, Masson’s trichrome staining and Oil red O staining were performed using specific commercial kits (Solarbio, Beijing, China).

### Immunofluorescence

To assess the ROS level in liver tissues, we conducted DHE staining. DHE powder was dissolved in PBS (20 mM) (Vigorous, Beijing, China). Following PBS washing of frozen liver tissue sections, they were incubated with a DHE dilution (2 μM). Subsequently, the sections were counterstained with DAPI. Images were captured, and analysis was performed using Image J (NIH, Bethesda, MD, USA). The resulting data was presented as relative fluorescence intensity.

### RNA transcriptome sequencing

Purified total RNA of mouse liver tissues was extracted using the TRIzol reagent (Invitrogen, CA, USA) according to the manufacturer’s guidelines. Using NanoDrop ND-1000, the amount and purity of RNA in each cardiac sample were measured (NanoDrop, DE). The integrity of the RNA was examined by a Bioanalyzer 2100 (Agilent, CA) and then validated by electrophoresis assay on a denaturated agarose gel. Utilizing Dynabeads Oligo (dT)25-61005 (Thermo Fisher, CA), poly (A) RNA is isolated from 1 μg total RNA. The magnesium RNA Fragmentation Module (NEB, cat. e6150, MA) was then utilized to fragment poly(A) RNA into small fragments. SuperScriptTM II reverse transcriptase was then used to convert the cleaved RNA fragments into cDNA (Invitrogen, cat. 1896649, CA, USA). As per the manufacturer guidelines, the sequencing analysis was completed using an Illumina NovaseqTM 6000. After the whole transcriptome generation, all transcripts’ expression levels were quantified. mRNAs with differential expression were identified utilizing the following criteria: fold change < 0.5 or fold change > 2, with *p*-value < 0.05. Pathway enriched was analyzed using modEnrichr (https://maayanlab.cloud/modEnrichr/).

### Reverse transcription-quantitative PCR (RT-qPCR)

Cultured cells or liver tissue samples were lysed with Trizol (cat. no. 15596026, Thermo Fisher; CA, USA). Total RNA was extracted and separated using chloroform and isopropanol and purified with ethanol. Reverse transcription was performed using the PrimeScript™ RT reagent Kit (cat. no. DRR037A, Takara Bio Inc., Kusatsu, Japan). Quantitative PCR was performed using TB Green® Premix Ex Taq™ II (cat. no. RR820B, Takara Bio Inc.). Primers (Supplementary Table S1) were obtained from Sangon Biotech (Shanghai, China). mRNA levels were detected and normalized using *Actb* as the loading control.

### Western blot analysis

Cells or liver tissue samples were lysed using RIPA buffer (cat# P0013B; Beyotime Biological Technology, Shanghai, China). Total proteins were separated using 10% sodium dodecyl sulfate-polyacrylamide gel electrophoresis (SDS-PAGE) and then electro-transferred to polyvinylidene fluoride (PVDF) membranes (cat. no. 1620177, Bio-Rad Laboratory; Hercules, CA). Membranes were blocked in 5% non-fat milk for 2 h at room temperature and cut into bands, followed by incubation with respective primary antibodies. After overnight incubation, protein bands were incubated with HRP-conjugated secondary antibodies and scanned using an image analyzer (Quantity One System; Bio-Rad, Richmond, CA, USA).

### Statistical analysis

All data were presented as mean ± SEM. Prism 8.0 software (GraphPad, San Diego, CA, USA) was used for the statistical analysis. Shapiro–Wilk test was used to assess the normality of our data distribution. Student’s *t* test was used to compare two groups of data. One-way ANOVA followed by Dunnett’s *post-hoc* test was used to compare more than two groups of data. A *p* value < 0.05 was considered as significant.

## Results

### Ala attenuates HFD-induced liver dysfunction

To evaluate the pharmacological effects of Ala, we used an HFD-induced obesity mouse model. Compared with the HFD group, Ala administration did not change body weight in the mice (Fig. 1B). However, Ala reduced serum ALT and AST levels in HFD-fed mice, indicating a hepatoprotective effect of Ala in HFD-fed mice (Fig. 1C). In addition, Ala treatment attenuated HFD-induced liver weight gain (Fig. 1D). Recent study has shown that regression of fatty liver is associated with favorable changes in blood lipids, and a decrease in BMI is independently associated with improvement in blood lipids [15]. Consistent with these studies, we found that Ala reduced serum levels of triglycerides (TG), total cholesterol (TCH), and low-density lipoprotein (LDL) in obese mice (Fig. 1E–G), indicating that Ala has the potential in regulating lipid metabolism in vivo. Then, the histological staining showed a significant increase in lipid droplets in the liver tissues of obesity mice. However, treatment with Ala reduced the accumulation of lipid droplets induced by HFD (Fig. 1H). To better quantify this effect, Oil red O staining was further used to evaluate the lipid accumulation in the livers of HFD-fed mice. Similar to the data of H&E staining, Ala treatment decreased the volume of lipid droplets in the liver (Fig. 1I) and the overall positive area (Supplementary Fig. S1A). *Fasn*, *Acaca* and *Srebp1* are genes related to fatty acid synthesis [16]. Thus, we measured the effect of Ala in the liver tissues of HFD-treated mice. Our data showed that Ala treatment significantly reduced the transcription of *Fasn*, *Acaca* and *Srebp1* in liver tissues, suggesting the potential role of Ala in regulating HFD-induced lipid metabolism (Supplementary Fig. S1B). Taken together, these results suggested that Ala reduced HFD-induced liver dysfunction.

### Ala attenuates HFD-induced liver fibrosis and collagen deposition

**Fig. 2:**
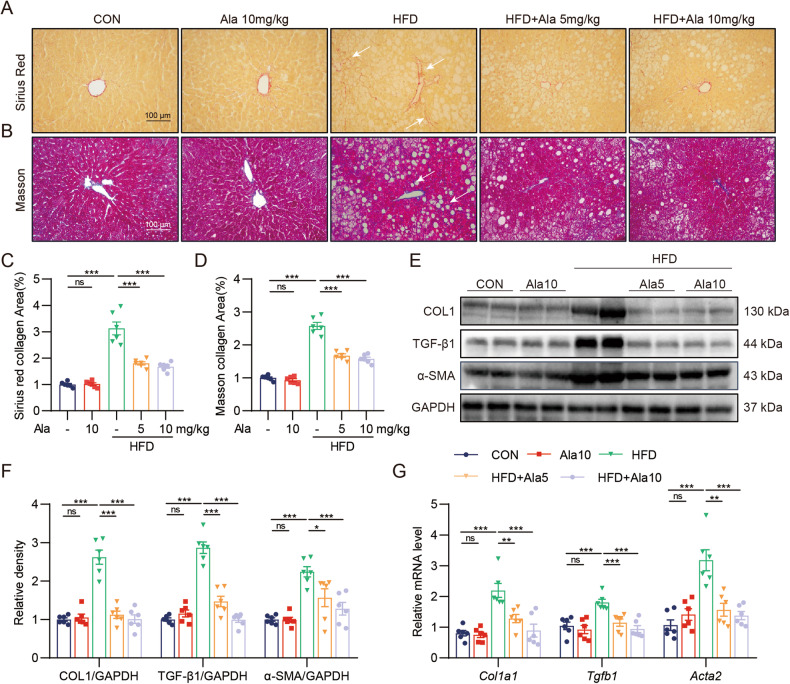
Ala attenuates HFD-induced liver fibrosis and collagen deposition. **A**, **B** Sirius red and Masson’s trichrome staining were performed. **C**, **D** Quantification of positive collagen area in Sirius red and Masson’s staining results. **E**, **F** Measurement of fibrosis-related proteins (COL1, TGF-β1 and α-SMA) in liver tissue lysates. Representative immunoblots (**E**) and densitometric quantification (**F**) are shown. **G** The mRNA levels of *Col1a1*, *Tgfb1* and *Acta2* were detected by RT-qPCR in the liver tissues. Transcripts were normalized to *Actb*. Data are expressed as mean ± SEM, *n* = 6 per group. *p < 0.05, **p < 0.01, ***p < 0.001, ns not significant.

With the pathological progression of NAFLD, liver fibrosis gradually occurs [17]. Sirius red and Masson trichrome staining were used to evaluate liver fibrosis. Our data showed a significant increase in total collagen content, indicating enhanced liver fibrosis in HFD-fed mice. Importantly, Ala significantly reduced these liver pathological changes (Fig. 2A–D). In addition, levels of fibrosis-associated proteins, such as COL1, TGF-β1 and α-SMA were reduced by Ala pretreatment in HFD mice, demonstrating the protective role of Ala in NAFLD (Fig. 2E, F). Similarly, RT-qPCR analysis also revealed that the transcription levels of *Col1a1, Tgfb1, and Acta2* in the liver of HFD-fed mice were decreased after treatment with Ala (Fig. 2G). Hence, these results implied that Ala inhibited HFD-induced liver fibrosis.

### Ala attenuates HFD-induced liver injuries by regulating the Nrf2/Keap1 pathway

**Fig. 3:**
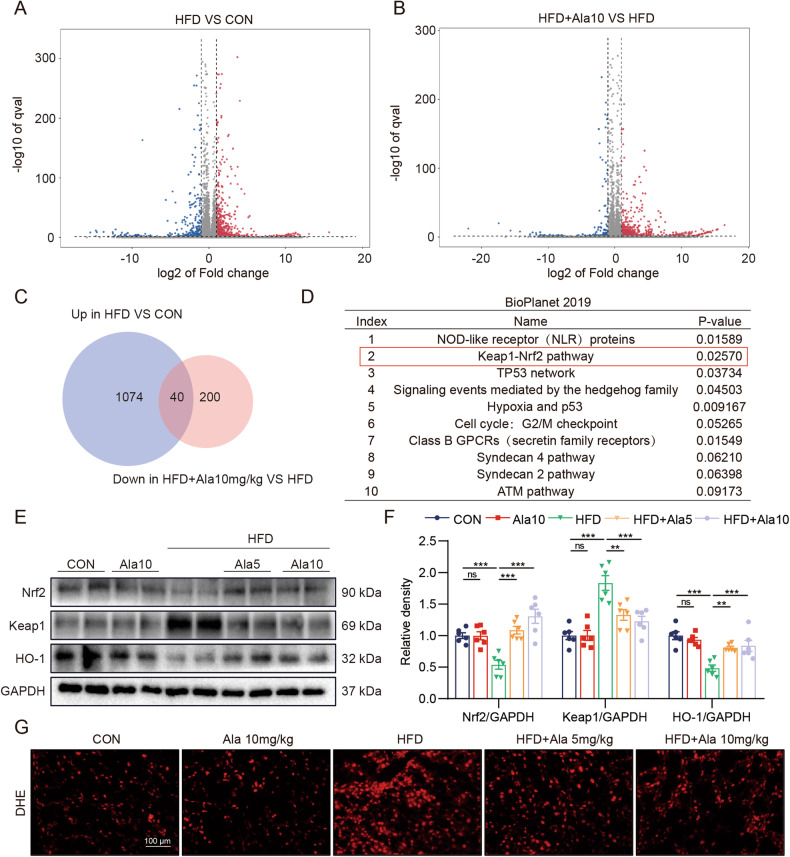
Ala attenuates HFD-induced liver injuries by regulating the Nrf2/Keap1 pathway. **A**, **B** Volcano map of differentially expressed genes between the HFD–treated group and the control group (**A**); Volcano map of differentially expressed genes between the HFD + Ala10 mg/kg group and the HFD group (**B**); the red, blue, and gray colors indicate the upregulated genes, the downregulated genes, and the genes with no difference, respectively. **C** Venn diagram of up-regulated genes of in HFD group vs CON group and down-regulated genes of in Ala10 mg/kg pretreatment + HFD group vs HFD group. **D** The list of BioPlanet 2019 pathway analysis followed by expression analysis of intersecting genes in plane C (a *P*-value using the Fisher exact test). **E** Representative Western blot analysis of Nrf2, Keap1 and HO-1 in the liver tissues. GAPDH was used as the loading control. **F** Quantitative data of the blot intensity of corresponding proteins determined by Image J. **G** Representative images of ROS staining in liver tissues [scale bar = 100 μm]. Data are expressed as mean ± SEM, *n* = 6 per group. ***p < 0.001, ns not significant.

RNA sequencing of mouse liver tissue was extracted to explore the protective mechanism of Ala on HFD-induced liver injury. A total of 1446 genes were significantly different between HFD and control mice, of which 1074 were upregulated and 372 were downregulated (Fig. 3A). In addition, a total of 912 genes were significantly different between the HFD + Ala 10 mg/kg group and HFD mice, of which 712 genes were upregulated and 200 genes were downregulated (Fig. 3B). 40 genes were controlled by Ala in the HFD environment (Fig. 3C). Interestingly, BioPlanet 2019 was used to analyze the expression of these 40 genes and found the possible involvement of Nrf2/Keap1 pathway (Fig. 3D). Nrf2/Keap1 signaling pathway, ranked top 2^nd^, has been reported to be associated with NAFLD [18]. It has been shown in various studies that the Nrf2/Keap1 pathway is the potential pathway for the treatment of NAFLD [19, 20]. As shown in Fig. 3E, F, the protein levels of Nrf2 and HO-1 were decreased while Keap1 was significantly increased in the HFD liver. However, Ala treatment reversed these changes. The mRNA levels of *Nfe2l2, Nqo-1* and *Hmox-1* were upregulated by Ala treatment (Supplementary Fig. S2A). Meanwhile, DHE staining results showed that ROS levels were increased in the liver tissues of obese mice, while Ala treatment reversed HFD-induced ROS production (Fig. 3G). Ala also enhanced the enzyme activity of SOD and decreased the levels of MDA in HFD‐induced mice (Supplementary Fig. S2B, C). These results suggested that the hepatoprotective ability of Ala might be related to the reduction of ROS production and activation of the Nrf2 pathway.

### Ala reduces inflammatory responses by inhibiting HFD-mediated NF-κB pathway

**Fig. 4:**
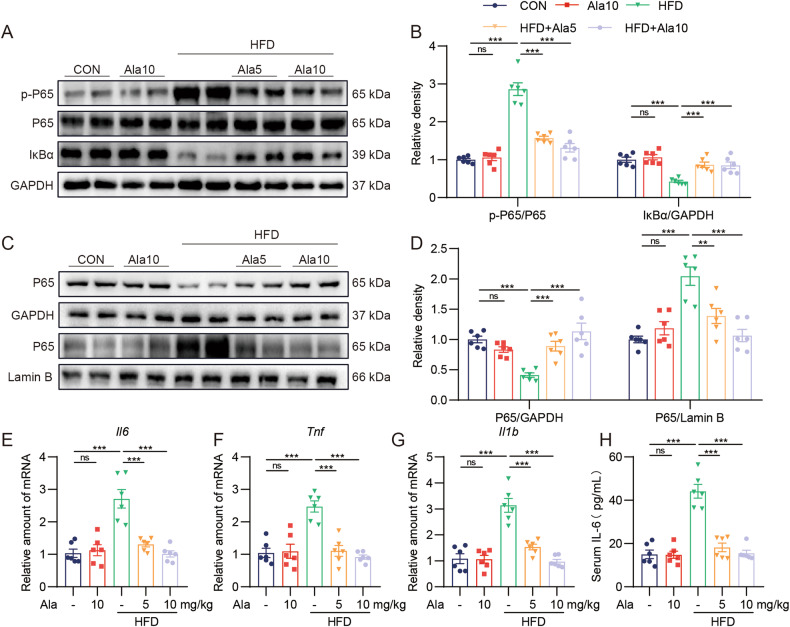
Ala reduces inflammatory responses by inhibiting HFD-mediated NF-κB pathway. **A**, **B** Representative immunoblot for p-P65, P65, IκB-α and GAPDH in liver tissues of mice (**A**). Densitometric quantification was performed using ImageJ (**B**). **C** Representative Western blot analysis of P65 in the liver tissues. GAPDH and Lamin B were used as the loading control. **D** Quantitative data of the blot intensity of corresponding proteins determined by Image J. **E**–**G** Relative mRNA levels of *Tnf*, *Il6* and *Il1b* in liver tissues. Transcripts were normalized to *Actb*. **H** Serum IL-6 protein levels. Data are expressed as mean ± SEM, *n* = 6 per group. **p < 0.01, ***p < 0.001.

NAFLD is characterized by ongoing inflammatory processes [21]. To clarify the role of Ala in HFD-induced liver inflammation, we examined the activation of the NF-κB signaling pathway. NF-κB signaling, a classical inflammatory pathway, is also activated in injured hepatocytes, thereby upregulating the transcription of inflammatory cytokines [5]. Compared with the CON group, the protein level of phosphorylated P65 was increased and the expression of IκB was decreased in the HFD group, suggesting that hyperlipidemia activated the NF-κB signaling pathway in the liver of obese mice, while Ala treatment significantly inhibited the activation of NF-κB pathway (Fig. 4A, B). As shown in Fig. 4C, D, the Ala treatment significantly inhibited HFD-induced p65 nuclear translocation in liver tissues. Similarly, we found that Ala treatment reversed HFD-induced increased mRNA levels of *Tnf*, *Il6* and *Il1b* (Fig. 4E–G). Moreover, HFD-induced an increase in serum IL-6 levels in mice, whereas Ala decreased this effect (Fig. 4H). Overall, Ala mitigated HFD-induced liver inflammation by inhibiting NF-κB activation.

### Ala reduces PA‐induced oxidative stress in AML-12 cells

**Fig. 5:**
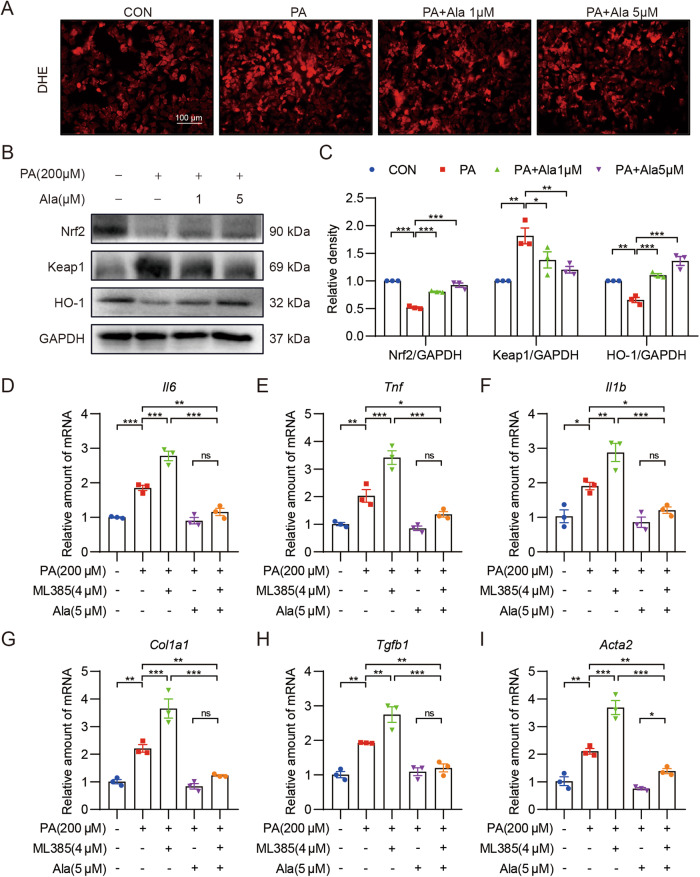
Ala inhibits PA-induced oxidative stress in AML-12 cells. **A** Representative images of ROS staining in AML cells. Cells with red fluorescence indicated intracellular ROS. **B**, **C** Western blot analysis was used to determine the protein levels of Nrf2, Keap1 and HO-1. GAPDH was used as the loading control. **D**–**F** Cells were pretreated with Ala with or without pretreatment of ML385, followed by exposure of PA for 12 h. The mRNA levels of *Tnf*, *Il6* and *Il1b* were measured via RT-qPCR. **G**–**I** AML cells were pretreated with Ala with or without pretreatment of ML385, followed by exposure of PA for 24 h. The mRNA levels of *Col1a1*, *Tgfb1* and *Acta2* were measured via RT-qPCR. Data are expressed as mean ± SEM, *n* = 3 per group. *p < 0.05, **p < 0.01, ***p < 0.001.

To confirm our in vivo findings, a palmitate acid (PA)-challenged hepatocyte model was used. Firstly, the optimal dose of Ala was first determined using MTT assay (Supplementary Fig. S3A). Doses of 1 and 5 μM Ala were selected as appropriate doses for further in vitro studies. We next investigated the effect of Ala on PA‐induced antioxidative response in AML-12 cells. As shown in Fig. 5A, DHE staining showed that increased ROS levels caused by PA were markedly reduced by pretreatment with Ala. In addition, Western blotting data revealed that PA treatment decreased Nrf2 and HO-1 expression and increased Keap1 expression in AML12 cells, indicating impairment of the antioxidant defense system. However, pretreatment with Ala reversed the phenotypes described above (Fig. 5B, C). Similarly, the mRNA levels of *Nfe2l2*, *Nqo-1* and *Hmox-1* were also upregulated by Ala treatment (Supplementary Fig. S3B). Furthermore, to clarify the potential link between the anti-inflammatory and antioxidant effects of Ala, a Nrf-2 inhibitor ML385 was used in our experiment [22]. RT-qPCR analysis revealed that ML385 exacerbated the inflammatory response, while also attenuating the anti-inflammatory effect of Ala (Fig. 5D–F). As shown in Fig. 5G–I, ML385 aggravated PA-induced fibrosis and attenuated the protective effect of Ala. However, the hepatoprotective and anti-inflammatory effects of Ala persisted even when Nrf-2 was inhibited compared with PA group. These data suggested that Ala exerts its protective effects, at least in part, through activation of the Nrf-2 pathway.

### Ala inhibits PA-induced inflammation and fibrosis in AML-12 cells

**Fig. 6:**
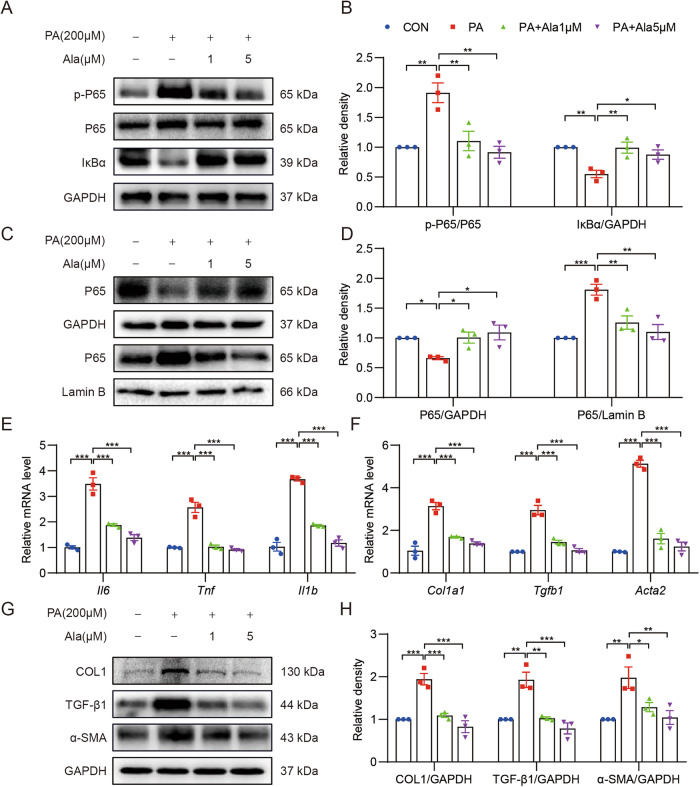
Ala reduces PA‐induced inflammation and fibrosis in AML-12 cells. **A**, **B** .Protein levels of p-P65, P65 and IκB-α were determined. AML cells were pretreated with Ala for 2 h, followed by exposure of PA for 1 h. Densitometric quantification was performed using ImageJ. **C**, **D** Representative Western blot analysis of P65 in AML cells. Densitometric quantification was performed using ImageJ. GAPDH and Lamin B were used as the loading control. **E** Cells were pretreated with Ala for 2 h, followed by exposure of PA for 12 h. The mRNA levels of *Tnf*, *Il6* and *Il1b* were measured via RT-qPCR. **F** AML cells were exposed to PA for 24 h after pretreatment with Ala for 2 h. mRNA levels of *Col1a1*, *Tgfb1*, and *Acta2* in PA-challenged AML cells. Transcripts were normalized to *Actb*. **G**, **H** The protein levels of COL1, TGF-β1 and α-SMA were detected in the cell lysis. The relative density of protein levels was quantified. Data are expressed as mean ± SEM, *n* = 3 per group. *p < 0.05, **p < 0.01, ***p < 0.001.

We have demonstrated the anti-inflammatory effect of Ala on NAFLD in vivo. Next, we examined the anti-inflammatory effect of Ala in AML cells. Western blot results showed that PA induced p65 phosphorylation and more IκBα degradation, which were reduced by Ala pretreatment (Fig. 6A, B). Also, as shown in Fig. 6C, D, Western blot analysis demonstrated that Ala pretreatment reduced the increase in nuclear p65 subunit levels induced by PA. In addition, the increased mRNA levels of proinflammatory cytokines such as *Tnf*, *Il6*, and *Il1b* induced by PA were reversed by Ala pretreatment (Fig. 6E). Our results also showed that hyperlipidemia increased the level of interleukin (IL-6) in media, which was alleviated by Ala pretreatment (Supplementary Fig. S4). Furthermore, the Western blotting and RT-qPCR data showed that Ala suppressed PA-increased mRNA and protein levels of COL1, TGF-β1 and α-SMA in AML-12 cells (Fig. 6F–H). Taken together, these results implied that Ala attenuated PA-induced fibrosis and inflammation in AML-12 cells.

## Discussion

In this study, we examined the potential therapeutic effects of Ala on NAFLD. Our study showed that Ala alleviated liver fibrosis and inflammation in HFD mice. Furthermore, our data indicated that the protective effect of Ala is achieved through regulating Nrf2/Keap1 pathway, as well as the suppression of HFD-induced NF-κB activation. More importantly, our study is the first to report the protective effect of Ala in NAFLD. In conclusion, Ala is expected to be a potential drug for the treatment of NAFLD.

NAFLD is a continuum of liver abnormalities ranging from nonalcoholic fatty liver disease (NAFL) to nonalcoholic steatohepatitis (NASH), with a variable course leading to cirrhosis and liver cancer [2]. Recent studies have shown that NAFLD is closely related to the excessive production of ROS, oxidative stress and inflammatory response in the liver [23]. Liver inflammation is a key event in the progression of NAFLD. Recent studies have shown that NAFLD is associated with substantial release and upregulation of proinflammatory mediators such as TNF-α, IL-6, and IL-1β [24]. Furthermore, ROS and inflammation-induced oxidative stress may be important mechanisms leading to hepatocyte death and tissue damage. Excessive accumulation of triglycerides aggravates the generation of ROS, which disrupts redox homeostasis by inducing oxidative stress and activates proinflammatory responses mediated by inflammatory signals [25, 26]. Conversely, activation of inflammatory signaling pathways can also lead to elevated ROS levels. The positive feedback of inflammation and oxidative stress accelerates the deterioration of NAFLD [27, 28]. Therefore, reducing oxidative stress and inflammatory response may be a therapeutic approach to treat NAFLD.

Despite the high prevalence of NAFLD, no pharmacologic therapies are currently approved for the specific treatment of NAFLD. Pharmacological antidiabetic, lipid-lowering, and natural bile acid therapy have been used to treat NAFLD but have disadvantages [29]. Therefore, it is of great practical significance to strengthen the research on the pathogenesis of NAFLD and find safe and effective drugs to prevent and treat NAFLD. Several natural products have been shown to have therapeutic effects in NAFLD [16]. Ala, an allergenic sesquiterpene lactone, has been reported to have a variety of pharmacological activities, such as anti-inflammatory, hepatoprotective, antifungal, antibacterial, and antitumor activities [30]. One of the most remarkable pharmacological effects of Ala is anti-inflammatory activity, which has been demonstrated in cellular and animal models of inflammation [12]. For instance, Ala can inhibit the neuroinflammatory response induced by middle cerebral artery occlusion/reperfusion (MCAO/R) in rats by inhibiting NF-κB activation [31]. Recent study indicated a protective effect of Ala on dextran sulfate sodium (DSS)-induced mice colitis possibly via PXR-mediated suppression of the NF-κB inflammatory signaling [32]. However, we have not yet found any research reports on the association between Ala and liver inflammatory diseases. Our study emphasized the potential use of Ala in the treatment of liver conditions. Also, our study represented the first attempt to investigate the effects of Ala on NAFLD. We found that Ala not only inhibited HFD-induced NF-κB activation but also induced the activation of the Nrf2-mediated antioxidant defense system. Therefore, our study found that Ala prevented NAFLD by inhibiting inflammation and oxidative stress.

However, our research does have certain limitations. First of all, we have yet to further investigate the target of Ala. Recent study has shown that Ala inhibits NLRP3 inflammasome activity by directly targeting the NACHT domain of NLRP3 [33]. In addition, Ala plays an anti-inflammatory role in LPS-stimulated RAW 264.7 cells by inhibiting NF-κB activation and MAPKs phosphorylation through down-regulating MyD88 signaling pathway [34]. Unfortunately, we did not observe the NF-κB pathway in BioPlanet 2019. We speculated that this might be due to a low gene intersection count and insufficient sample size, resulting in a lack of differences in this pathway. Additional research is required to validate the potential target of Ala and elucidate the precise molecular mechanism in our study. Secondly, we did not conduct glucose and insulin tolerance related experiments, which is a limitation of our study that requires further investigation in the future. Although we did not measure these data, other studies have revealed related findings. For example, Kim et al. has found that Ala inhibits IL-6-induced skeletal muscle inflammation via alleviating glucose intolerance and insulin resistance [35]. In another study, Ala improves palmitate-induced glucose intolerance and inflammation in both lean and obese states in vitro [36]. Besides, we hypothesize that Ala will also be effective in the high sucrose diet model. However, further experiments are needed to confirm the effect of Ala in a model undergoing high sucrose diet. Thirdly, the mouse model we selected has certain limitations in exploring liver fibrosis. Although this is not the most classical model, it is still a well-accepted model for the study of NAFLD [37, 38]. Also, our data show that HFD-caused marked liver fibrosis and collagen deposition, as indicated by the data of Sirius red and Masson trichrome staining. In addition, levels of fibrosis-associated proteins, such as COL1, TGF-β1 and α-SMA, were significantly increased in livers of HFD-fed mice. All these data suggest that HFD-fed mouse model develop significant liver fibrosis in our study. In the next phase, we will further explore the impact of Ala on liver fibrosis in CCl_4_-induced model. Lastly, we did not collect adipose tissue to evaluate the effect of Ala affecting tissue lipolysis, which is a limitation of our study. However, one study reported that Ala inhibites JNK activation in 3T3-L1 adipocytes through inhibition of TLR4-JNK pathway and preventes palmitate-induced fat accumulation in 3T3-L1 adipocytes [36]. These results imply that Ala may also have an effect on adipose tissue. Nonetheless, our study focused more on the protective effect of Ala against HFD-induced liver inflammation.

In addition, Ala may protect the liver by modulating other signaling pathway. For example, RNA-seq analysis suggested that Ala might also regulate the NOD-like signaling pathway. Studies have shown that a high-fat diet causes intestinal leakage and activates NOD1 and NOD2 in organs [39]. Also, the expression of both NOD1 and NOD2 was upregulated in HFD mice [40]. Following HFD feeding, *Nod1*^−/−^ mice were protected against the development of metabolic syndrome, while *Nod2*^−/−^ mice fed a HFD had aggravated inflammation and obesity, as well as increased insulin resistance [41, 42]. NOD1 and NOD2 have been demonstrated to directly connect endoplasmic reticulum (ER) stress with inflammation across various cell types, although the exact mechanisms responsible for this connection are not fully elucidated [40]. In a previous study, Troxerutin to some extent reduces the expression of NOD1 and NOD2 and diminishes the signaling of NOD1 and NOD2 by disrupting their functional interaction with the kinase RIP. This ultimately leads to decreased expression of hepatic inflammatory factors [43]. Therefore, NOD like signaling pathway might be a potential pathway for Ala treatment of NAFLD as well.

## Conclusion

In summary, our findings demonstrated that Ala confers protection against NAFLD by effectively inhibiting inflammation and oxidative stress, while also mitigating liver fibrosis. This multifaceted beneficial impact of Ala is closely associated with its capacity to suppress NF-κB-mediated inflammation and regulate the Nrf2/Keap1 pathway, thereby fostering anti-oxidative effects. These results, validated both in vivo and in vitro, strongly advocated for the consideration of Ala as a promising therapeutic candidate in the treatment of NAFLD.

### Supplementary information

Supplyfile

Supplyfile

## Data Availability

The data underlying this article will be shared on reasonable request to the corresponding author.

## References

[1] Fan JG, Kim SU, Wong VW (2017). New trends on obesity and NAFLD in Asia. J Hepatol.

[2] Friedman SL, Neuschwander-Tetri BA, Rinella M, Sanyal AJ (2018). Mechanisms of NAFLD development and therapeutic strategies. Nat Med.

[3] Ipsen DH, Lykkesfeldt J, Tveden-Nyborg P (2018). Molecular mechanisms of hepatic lipid accumulation in non-alcoholic fatty liver disease. Cell Mol Life Sci.

[4] Cobbina E, Akhlaghi F (2017). Non-alcoholic fatty liver disease (NAFLD) - pathogenesis, classification, and effect on drug metabolizing enzymes and transporters. Drug Metab Rev.

[5] Wang Q, Ou Y, Hu G, Wen C, Yue S, Chen C (2020). Naringenin attenuates non-alcoholic fatty liver disease by down-regulating the NLRP3/NF-kappaB pathway in mice. Br J Pharmacol.

[6] Jou J, Choi SS, Diehl AM (2008). Mechanisms of disease progression in nonalcoholic fatty liver disease. Semin Liver Dis.

[7] Browning JD, Horton JD (2004). Molecular mediators of hepatic steatosis and liver injury. J Clin Invest.

[8] Bell M, Wang H, Chen H, McLenithan JC, Gong DW, Yang RZ (2008). Consequences of lipid droplet coat protein downregulation in liver cells: abnormal lipid droplet metabolism and induction of insulin resistance. Diabetes.

[9] Xu J, Shen J, Yuan R, Jia B, Zhang Y, Wang S (2021). Mitochondrial Targeting Therapeutics: Promising Role of Natural Products in Non-alcoholic Fatty Liver Disease. Front Pharmacol.

[10] Farzanegi P, Dana A, Ebrahimpoor Z, Asadi M, Azarbayjani MA (2019). Mechanisms of beneficial effects of exercise training on non-alcoholic fatty liver disease (NAFLD): Roles of oxidative stress and inflammation. Eur J Sport Sci.

[11] Cai Y, Gao K, Peng B, Xu Z, Peng J, Li J (2021). Alantolactone: A Natural Plant Extract as a Potential Therapeutic Agent for Cancer. Front Pharmacol.

[12] Liu X, Bian L, Duan X, Zhuang X, Sui Y, Yang L (2021). Alantolactone: A sesquiterpene lactone with diverse pharmacological effects. Chem Biol Drug Des.

[13] Zhu Y, Ling Y, Wang X (2020). Alantolactone mitigates renal injury induced by diabetes via inhibition of high glucose-mediated inflammatory response and macrophage infiltration. Immunopharmacol Immunotoxicol.

[14] Dang X, He B, Ning Q, Liu Y, Guo J, Niu G (2020). Alantolactone suppresses inflammation, apoptosis and oxidative stress in cigarette smoke-induced human bronchial epithelial cells through activation of Nrf2/HO-1 and inhibition of the NF-kappaB pathways. Respir Res.

[15] Sung KC, Lee MY, Lee JY, Lee SH, Kim JY, Wild SH (2018). Resolution of fatty liver and weight loss: Independent associations with changes in serum lipids and apolipoproteins. Atherosclerosis.

[16] Guo X, Yin X, Liu Z, Wang J (2022). Non-Alcoholic Fatty Liver Disease (NAFLD) Pathogenesis and Natural Products for Prevention and Treatment. Int J Mol Sci.

[17] Ahmed H, Umar MI, Imran S, Javaid F, Syed SK, Riaz R (2022). TGF-beta1 signaling can worsen NAFLD with liver fibrosis backdrop. Exp Mol Pathol.

[18] Wang Y, Liu B, Wu P, Chu Y, Gui S, Zheng Y (2022). Dietary Selenium Alleviated Mouse Liver Oxidative Stress and NAFLD Induced by Obesity by Regulating the KEAP1/NRF2 Pathway. Antioxidants.

[19] Yu H, Yan S, Jin M, Wei Y, Zhao L, Cheng J (2023). Aescin can alleviate NAFLD through Keap1-Nrf2 by activating antioxidant and autophagy. Phytomedicine.

[20] Lee DH, Park JS, Lee YS, Han J, Lee DK, Kwon SW (2020). SQSTM1/p62 activates NFE2L2/NRF2 via ULK1-mediated autophagic KEAP1 degradation and protects mouse liver from lipotoxicity. Autophagy.

[21] Wiering L, Tacke F (2023). Treating inflammation to combat non-alcoholic fatty liver disease. J Endocrinol.

[22] Singh A, Venkannagari S, Oh KH, Zhang YQ, Rohde JM, Liu L (2016). Small Molecule Inhibitor of NRF2 Selectively Intervenes Therapeutic Resistance in KEAP1-Deficient NSCLC Tumors. ACS Chem Biol.

[23] Braud L, Battault S, Meyer G, Nascimento A, Gaillard S, de Sousa G (2017). Antioxidant properties of tea blunt ROS-dependent lipogenesis: beneficial effect on hepatic steatosis in a high fat-high sucrose diet NAFLD obese rat model. J Nutr Biochem.

[24] Hajighasem A, Farzanegi P, Mazaheri Z (2019). Effects of combined therapy with resveratrol, continuous and interval exercises on apoptosis, oxidative stress, and inflammatory biomarkers in the liver of old rats with non-alcoholic fatty liver disease. Arch Physiol Biochem.

[25] Masarone M, Rosato V, Dallio M, Gravina AG, Aglitti A, Loguercio C (2018). Role of Oxidative Stress in Pathophysiology of Nonalcoholic Fatty Liver Disease. Oxid Med Cell Longev.

[26] Li S, Hong M, Tan HY, Wang N, Feng Y (2016). Insights into the Role and Interdependence of Oxidative Stress and Inflammation in Liver Diseases. Oxid Med Cell Longev.

[27] Arroyave-Ospina JC, Wu Z, Geng Y, Moshage H (2021). Role of Oxidative Stress in the Pathogenesis of Non-Alcoholic Fatty Liver Disease: Implications for Prevention and Therapy. Antioxidants.

[28] Arrese M, Cabrera D, Kalergis AM, Feldstein AE (2016). Innate Immunity and Inflammation in NAFLD/NASH. Dig Dis Sci.

[29] Nassir F (2022). NAFLD: Mechanisms, Treatments, and Biomarkers. Biomolecules.

[30] Cui L, Bu W, Song J, Feng L, Xu T, Liu D (2018). Apoptosis induction by alantolactone in breast cancer MDA-MB-231 cells through reactive oxygen species-mediated mitochondrion-dependent pathway. Arch Pharm Res.

[31] Tan L, Li J, Wang Y, Tan R (2019). Anti-Neuroinflammatory Effect of Alantolactone through the Suppression of the NF-kappaB and MAPK Signaling Pathways. Cells.

[32] Ren Y, Yue B, Ren G, Yu Z, Luo X, Sun A (2019). Activation of PXR by alantolactone ameliorates DSS-induced experimental colitis via suppressing NF-kappaB signaling pathway. Sci Rep.

[33] Li W, Xu H, Shao J, Chen J, Lin Y, Zheng Z (2023). Discovery of alantolactone as a naturally occurring NLRP3 inhibitor to alleviate NLRP3-driven inflammatory diseases in mice. Br J Pharmacol.

[34] Chun J, Choi RJ, Khan S, Lee DS, Kim YC, Nam YJ (2012). Alantolactone suppresses inducible nitric oxide synthase and cyclooxygenase-2 expression by down-regulating NF-kappaB, MAPK and AP-1 via the MyD88 signaling pathway in LPS-activated RAW 264.7 cells. Int Immunopharmacol.

[35] Kim M, Song K, Kim YS (2017). Alantolactone Improves Prolonged Exposure of Interleukin-6-Induced Skeletal Muscle Inflammation Associated Glucose Intolerance and Insulin Resistance. Front Pharmacol.

[36] Kim M, Song K, Kim YS (2017). Alantolactone improves palmitate-induced glucose intolerance and inflammation in both lean and obese states in vitro: Adipocyte and adipocyte-macrophage co-culture system. Int Immunopharmacol.

[37] Lan T, Yu Y, Zhang J, Li H, Weng Q, Jiang S (2021). Cordycepin Ameliorates Nonalcoholic Steatohepatitis by Activation of the AMP-Activated Protein Kinase Signaling Pathway. Hepatology.

[38] Hou X, Yin S, Ren R, Liu S, Yong L, Liu Y (2021). Myeloid-Cell-Specific IL-6 Signaling Promotes MicroRNA-223-Enriched Exosome Production to Attenuate NAFLD-Associated Fibrosis. Hepatology.

[39] Omaru N, Watanabe T, Kamata K, Minaga K, Kudo M (2022). Activation of NOD1 and NOD2 in the development of liver injury and cancer. Front Immunol.

[40] Zangara MT, Johnston I, Johnson EE, McDonald C (2021). Mediators of Metabolism: An Unconventional Role for NOD1 and NOD2. Int J Mol Sci.

[41] Amar J, Chabo C, Waget A, Klopp P, Vachoux C, Bermudez-Humaran LG (2011). Intestinal mucosal adherence and translocation of commensal bacteria at the early onset of type 2 diabetes: molecular mechanisms and probiotic treatment. EMBO Mol Med.

[42] Denou E, Lolmede K, Garidou L, Pomie C, Chabo C, Lau TC (2015). Defective NOD2 peptidoglycan sensing promotes diet-induced inflammation, dysbiosis, and insulin resistance. EMBO Mol Med.

[43] Zhang Z, Wang X, Zheng G, Shan Q, Lu J, Fan S (2016). Troxerutin Attenuates Enhancement of Hepatic Gluconeogenesis by Inhibiting NOD Activation-Mediated Inflammation in High-Fat Diet-Treated Mice. Int J Mol Sci.

