# Supplementary figures and images for: Alantolactone attenuates high-fat diet-induced inflammation and oxidative stress in non-alcoholic fatty liver disease
Source: Nutr Diabetes. 2024 Jun 10;14:41. doi: 10.1038/s41387-024-00300-7 (PMC11164993; doi:10.1038/s41387-024-00300-7)

Fig.2E

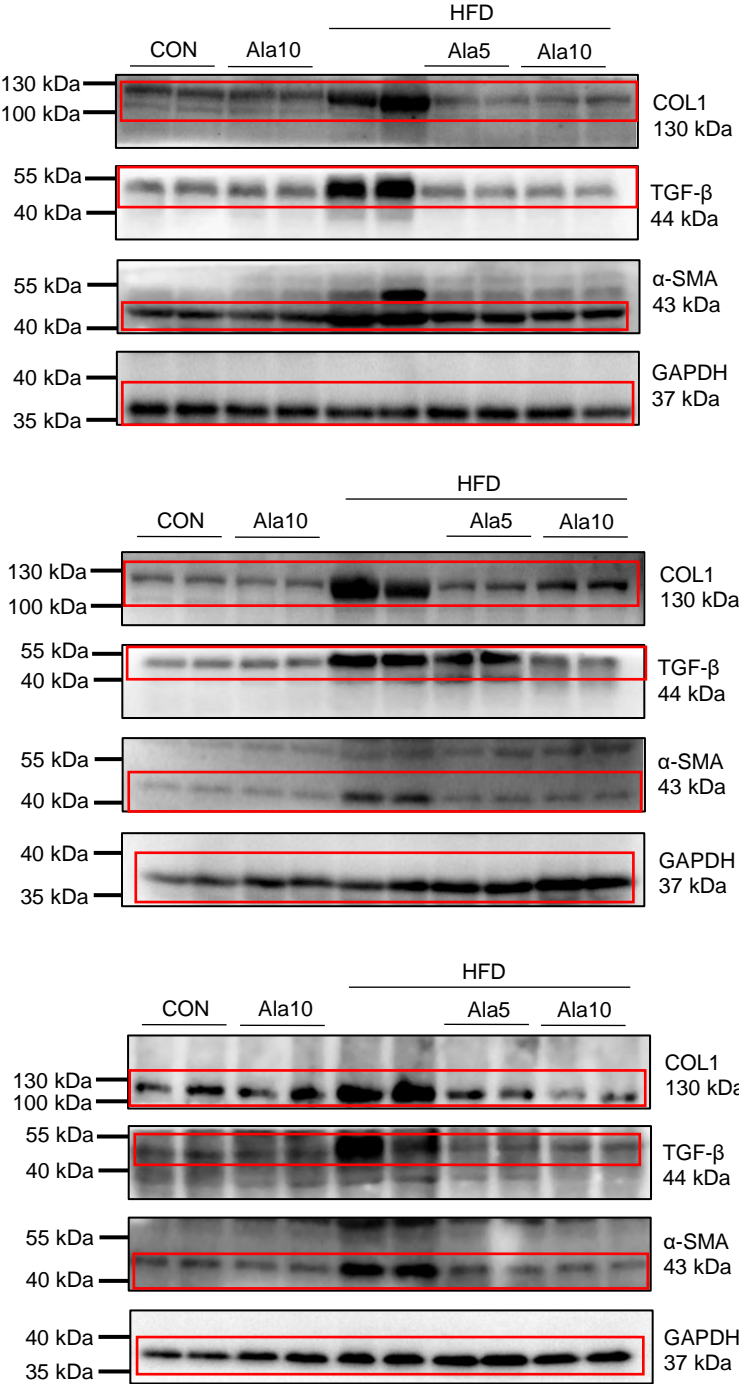

Fig.3E

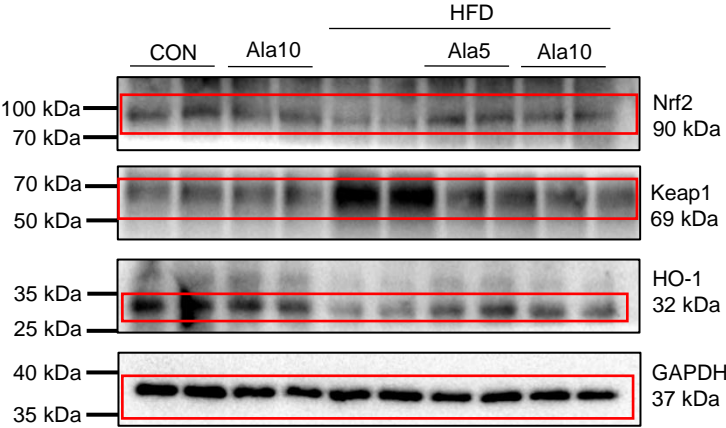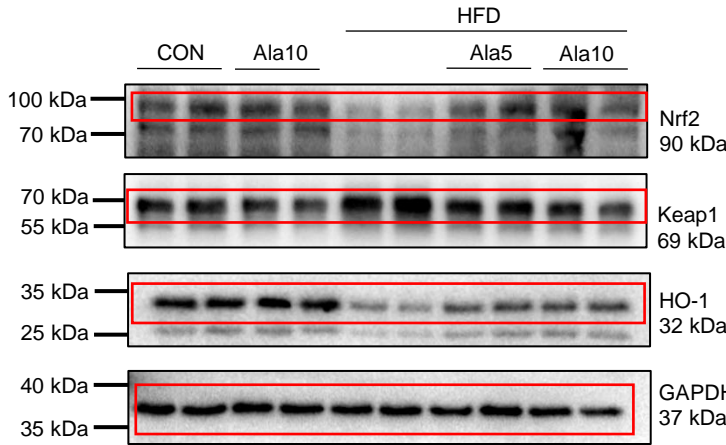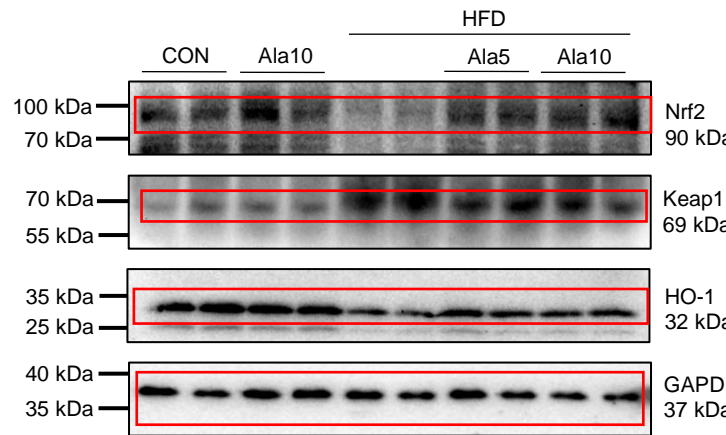

Fig.4A

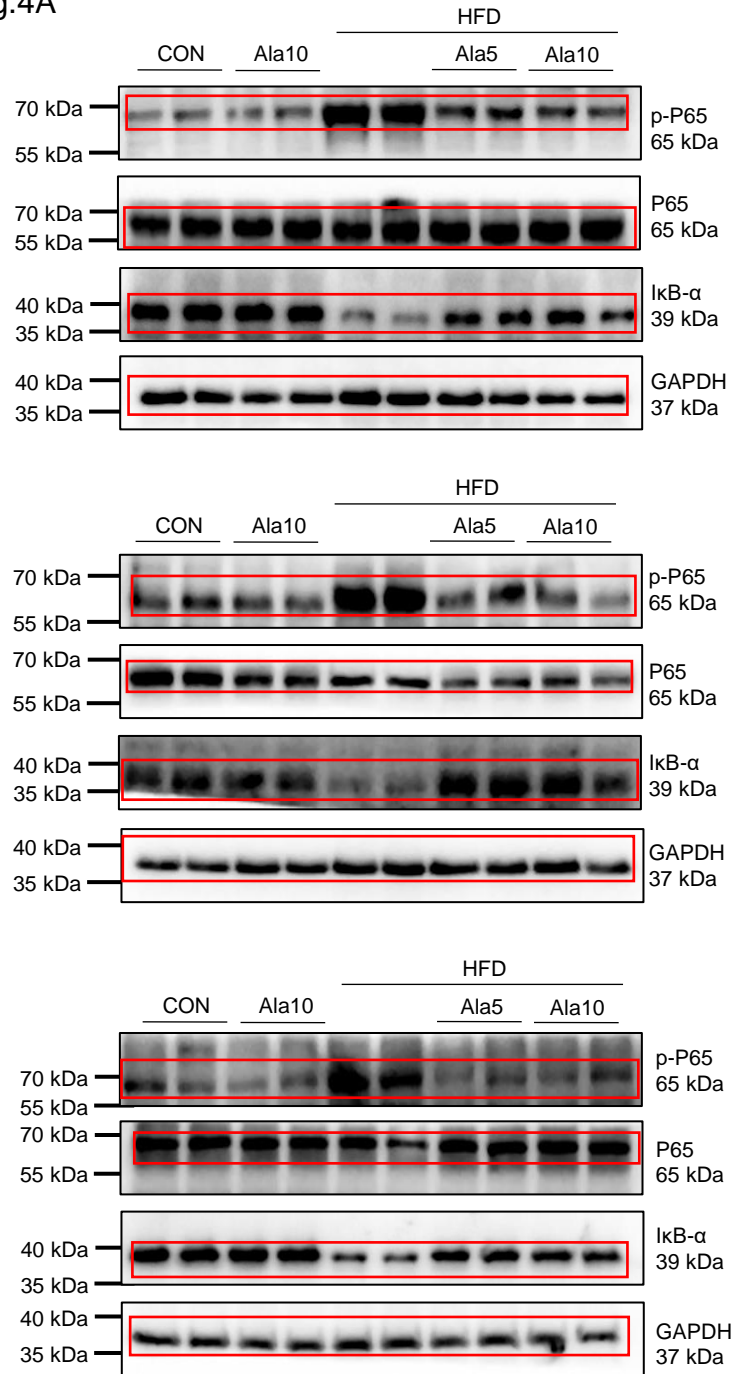

Fig.4C

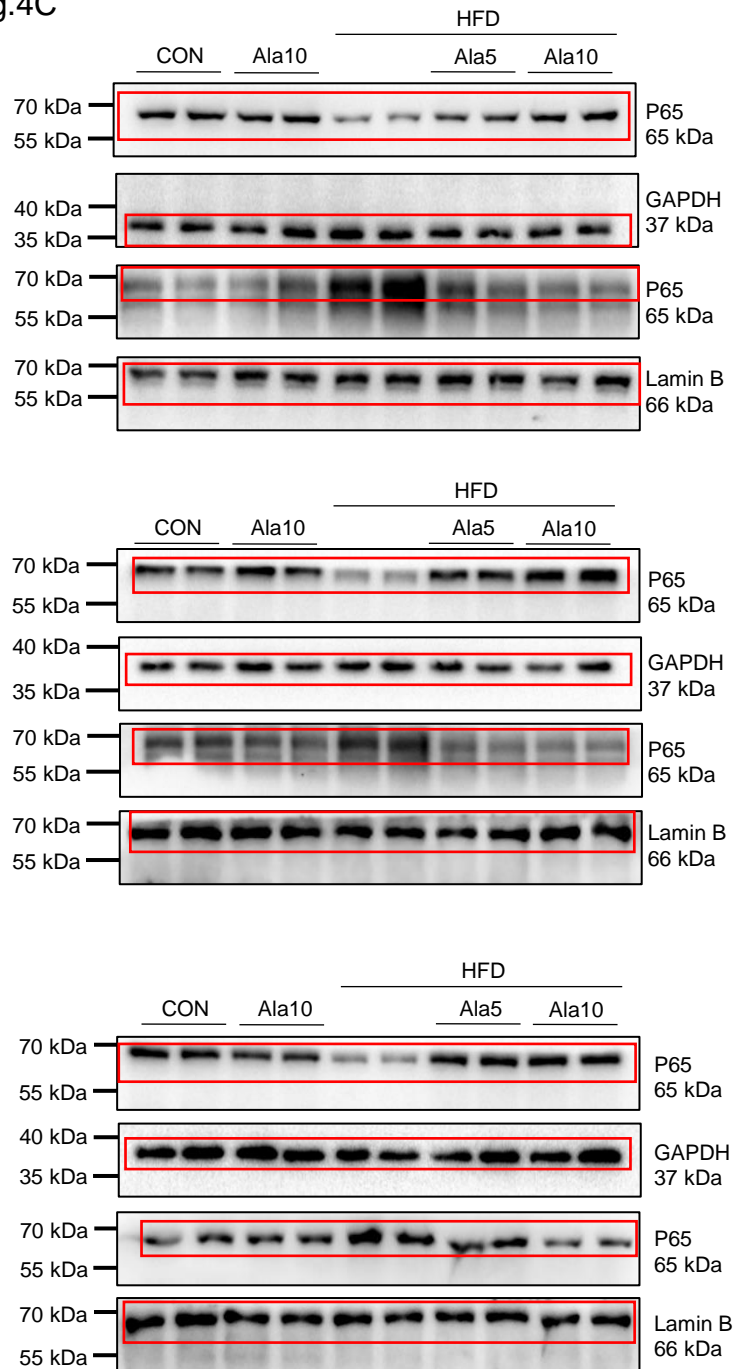

Fig.5B

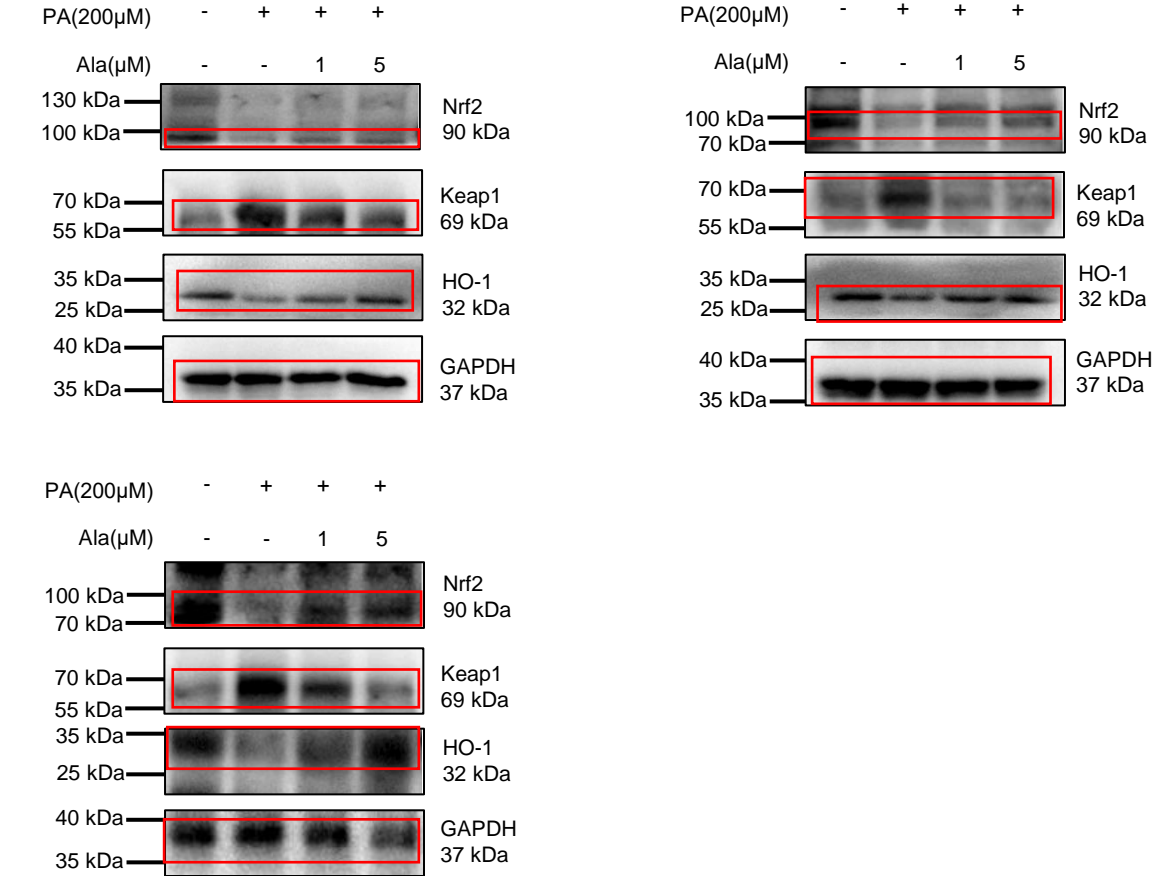

Fig.6A

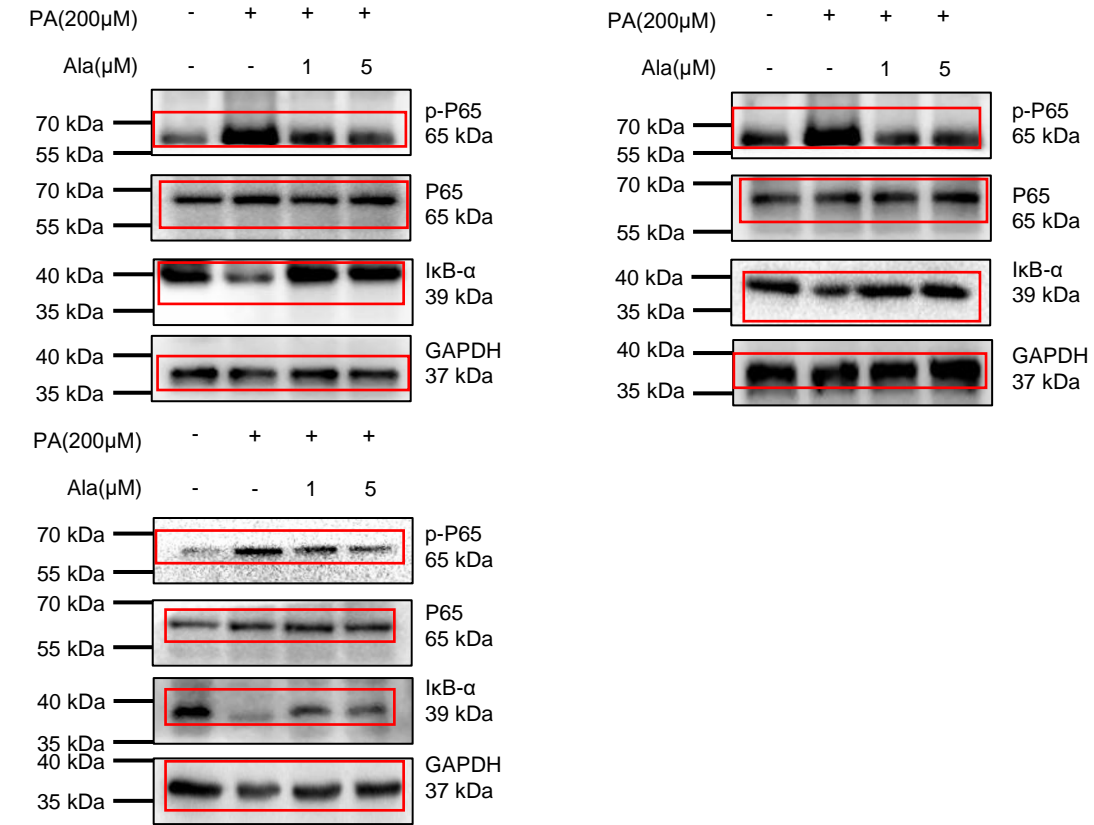

Fig.6C

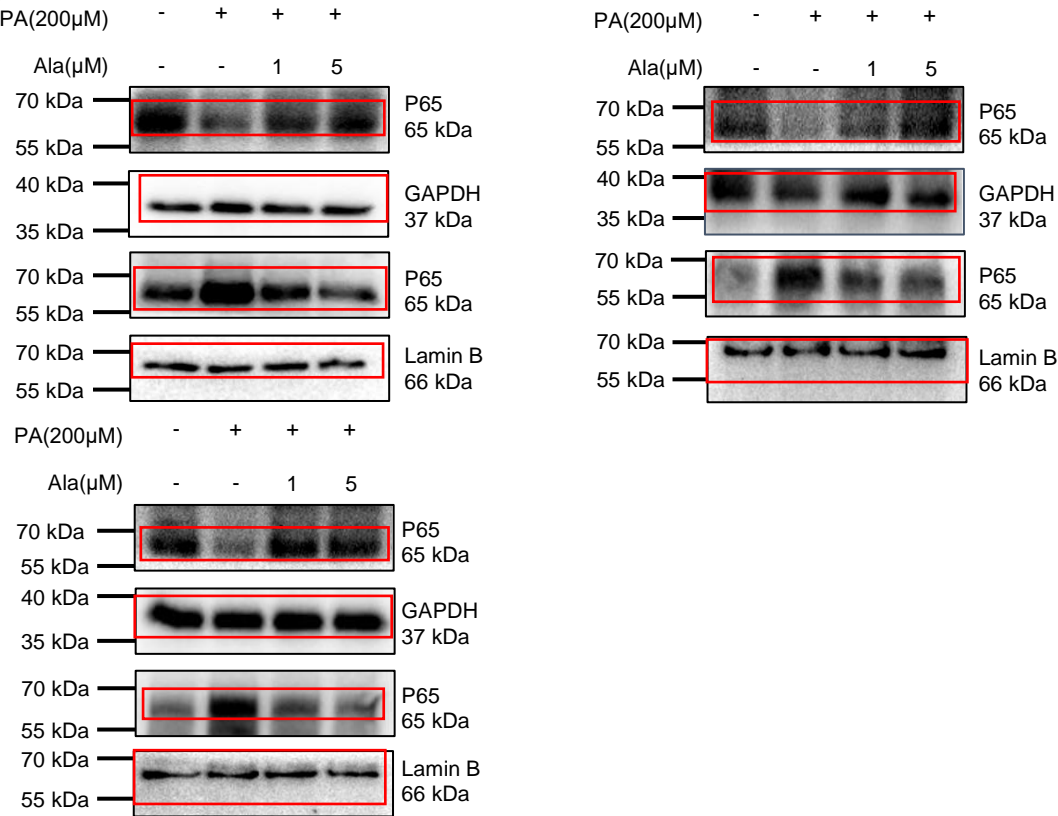

Fig.6G

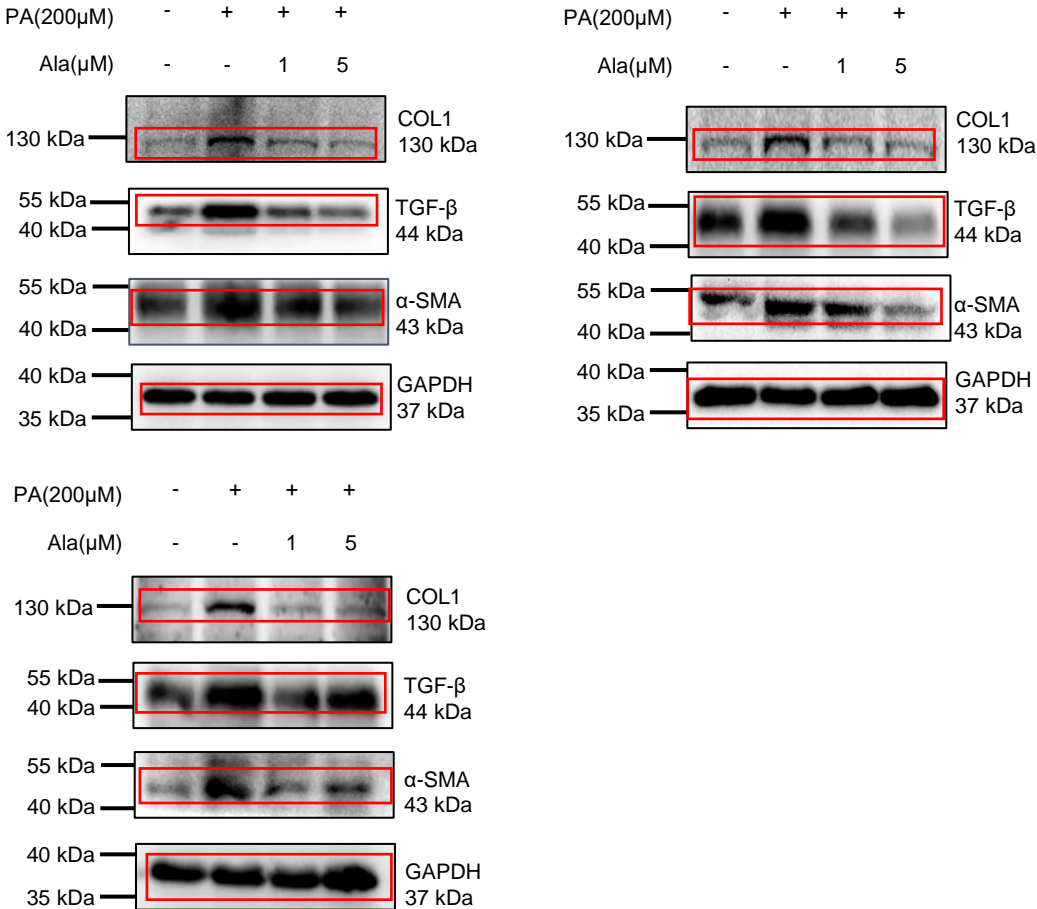

Supplement: Supplementary file 1 — Supplyfile [file 41387_2024_300_MOESM1_ESM.pdf]
