# Supplementary material for: Alantolactone attenuates high-fat diet-induced inflammation and oxidative stress in non-alcoholic fatty liver disease
Source: Nutr Diabetes. 2024 Jun 10;14:41. doi: 10.1038/s41387-024-00300-7 (PMC11164993; doi:10.1038/s41387-024-00300-7)
Supplement: Supplementary file 2 — Supplyfile [file 41387_2024_300_MOESM2_ESM.docx]

***Supplementary Materials and Methods***

**Alantolactone attenuates high-fat diet-induced inflammation and oxidative stress in non-alcoholic fatty liver disease**

Supplementary file contains 1 table and 3 figures.

**Supplementary Table S1.** Primer sequences for qPCR.

| Gene Name | Species | Primer sequence (5’-3’) | |
| --- | --- | --- | --- |
| *Actb* | Mouse | Forward | CCGTGAAAAGATGACCCAGA |
|  |  | Reversed | TACGACCAGAGGCATACAG |
| *Tgfb1* | Mouse | Forward | CTCCCGTGGCTTCTAGTGC |
|  |  | Reversed | GCCTTAGTTTGGACAGGATCTG |
| *Co11a1* | Mouse | Forward | AATGGTGCTCCTGGTATTGC |
|  |  | Reversed | GGTCCTCGTTTTCCTTCTT |
| *Acta2* | Mouse | Forward | GTCCCAGACATCAGGGAGTAA |
|  |  | Reversed | TCGGATACTTCAGCGTCAGGA |
| *Tnf* | Mouse | Forward  Reversed | CAGGGGCCACCACGCTCTTC  TTTGTGAGTGTGAGGGTCTGG |
| *Il6* | Mouse | Forward  Reversed | TAGTCCTTCCTACCCCAATTTCC  TTGGTCCTTAGCCACTCCTTC |
| *Il1b* | Mouse | Forward  Reversed | ACTCCTTAGTCCTCGGCCA  CCATCAGAGGCAAGGAGGAA |
| *Nfe2l2* | Mouse | Forward  Reversed | TCTTGGAGTAAGTCGAGAAGTGT  GTTGAAACTGAGCGAAAAAGGC |
| *Nqo-1* | Mouse | Forward  Reversed | AGGATGGGAGGTACTCGAATC  AGGCGTCCTTCCTTATATGCTA |
| *Hmox1* | Mouse | Forward  Reversed | GTTGAAACTGAGCGAAAAAGGC  GCCGTGTAGATATGGTACAAGGA |
| *Fasn* | Mouse | Forward  Reversed | TATCAAGGAGGCCCATTTTGC  TGTTTCCACTTCTAAACCATGCT |
| *Acaca* | Mouse | Forward  Reversed | GATGAACCATCTCCGTTGGC  GACCCAATTATGAATCGGGAGTG |
| *Srebp1* | Mouse | Forward  Reversed | GCAGCCACCATCTAGCCTG  CAGCAGTGAGTCTGCCTTGAT |

**
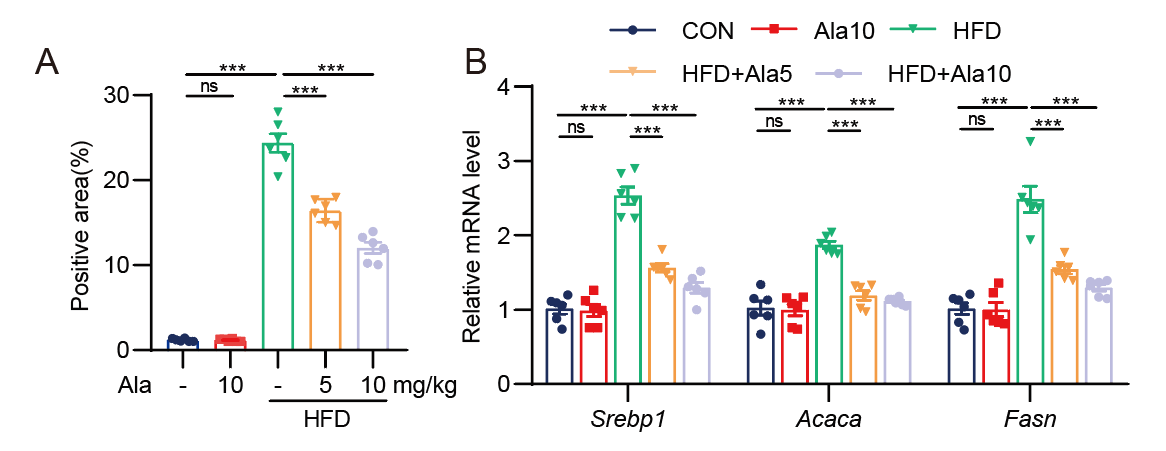
**

**Supplementary Figure S1. Ala alleviates HFD-induced lipid accumulation.** (A) Statistical diagram of positive areas stained with Oil red O. (B) mRNA levels of *Srebp1*, *Acaca* and *Fasn* in liver tissues were determined using RT-qPCR. Transcripts were normalized to *Actb*. Data are expressed as mean ± SEM, n=6 per group. *** *p* < 0.001, ns = not significant.

**
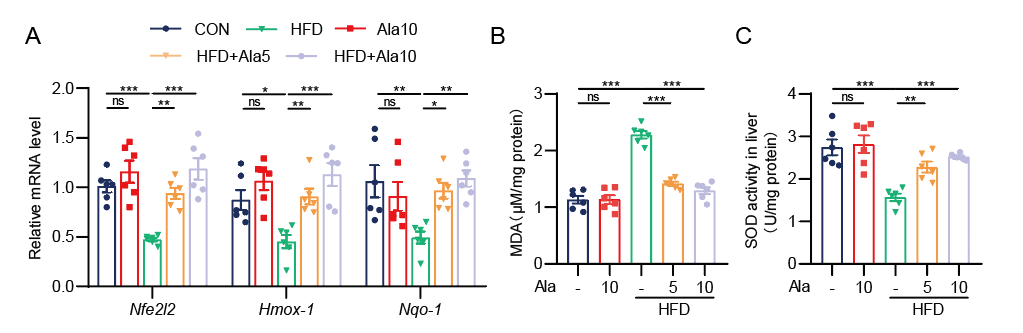
Supplementary Figure S2. Ala attenuates HFD-induced liver injuries by regulating the Nrf2/Keap1 pathway.** (A) mRNA levels of *Nfe2l2, Hmox-1* and *Nqo-1* in livers of mice determined by RT-qPCR. Transcripts were normalized to *Actb.* (B-C) MDA and SOD levels in mouse liver tissues were determined by ELISA kits. Data are expressed as mean ± SEM, n=6 per group. * *p* < 0.05, ** *p* < 0.01, *** *p* < 0.001, ns = not significant.


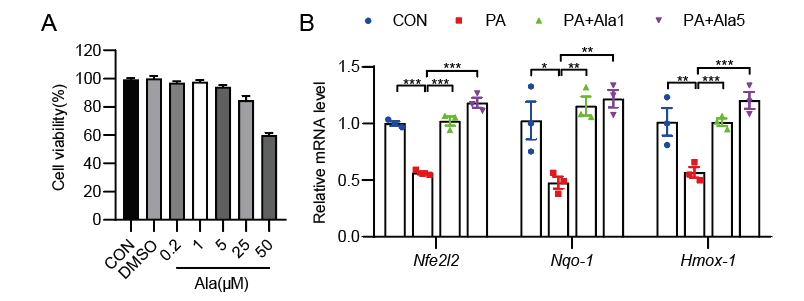


**Supplementary Figure S3. Ala reduces PA‐induced oxidative stress in AML-12 cells.** (A) The cytotoxicity of Ala in AML cells was determined using MTT assay. (B) mRNA levels of *Nfe2l2, Hmox-1* and *Nqo-1* in AML cells determined by RT-qPCR. Transcripts were normalized to *Actb.* Data are expressed as mean ± SEM, n=3 per group. * *p* < 0.05, ** *p* < 0.01, *** *p* < 0.001.

**
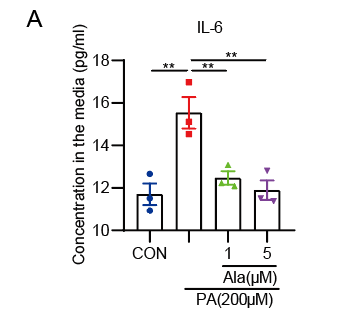
**

**Supplementary Figure S4.** **Ala inhibits PA-induced inflammation and fibrosis in AML-12 cells.** Serum IL-6 protein levels. Data are expressed as mean ± SEM, n=3 per group. ** *p* < 0.01.
